# Supplementary material for: Profilin Pfy1 is critical for cell wall integrity and virulence in Candida albicans
Source: Microbiol Spectr. 2025 Feb 24;13(4):e02593-24. doi: 10.1128/spectrum.02593-24 (PMC11960436; doi:10.1128/spectrum.02593-24)
Supplement: Supplemental figures — Fig. S1 to S4. [file spectrum.02593-24-s0001.docx]

**
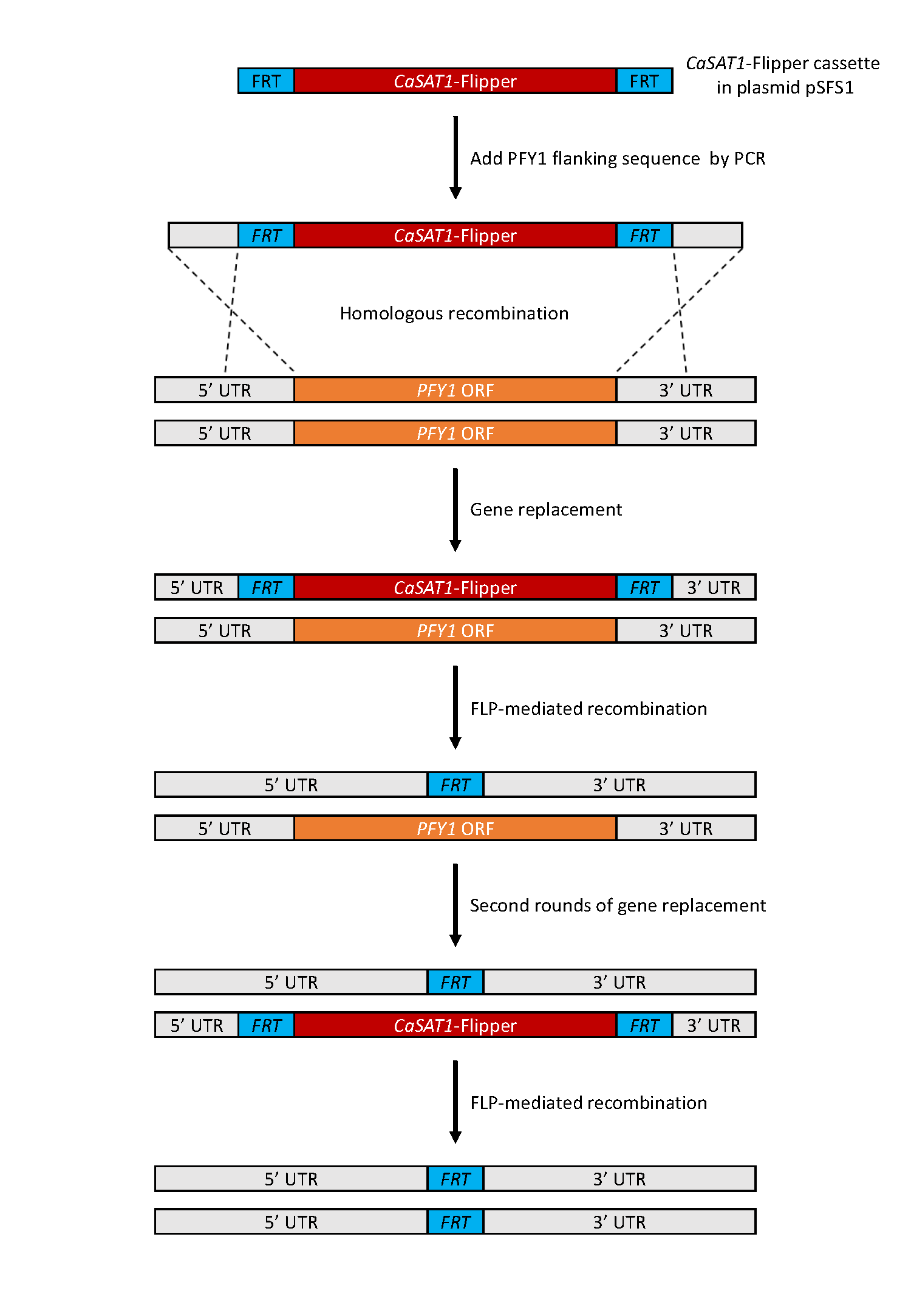
**

**Figure S1. Schematic diagram of the construction of *pfy1* mutant**

**
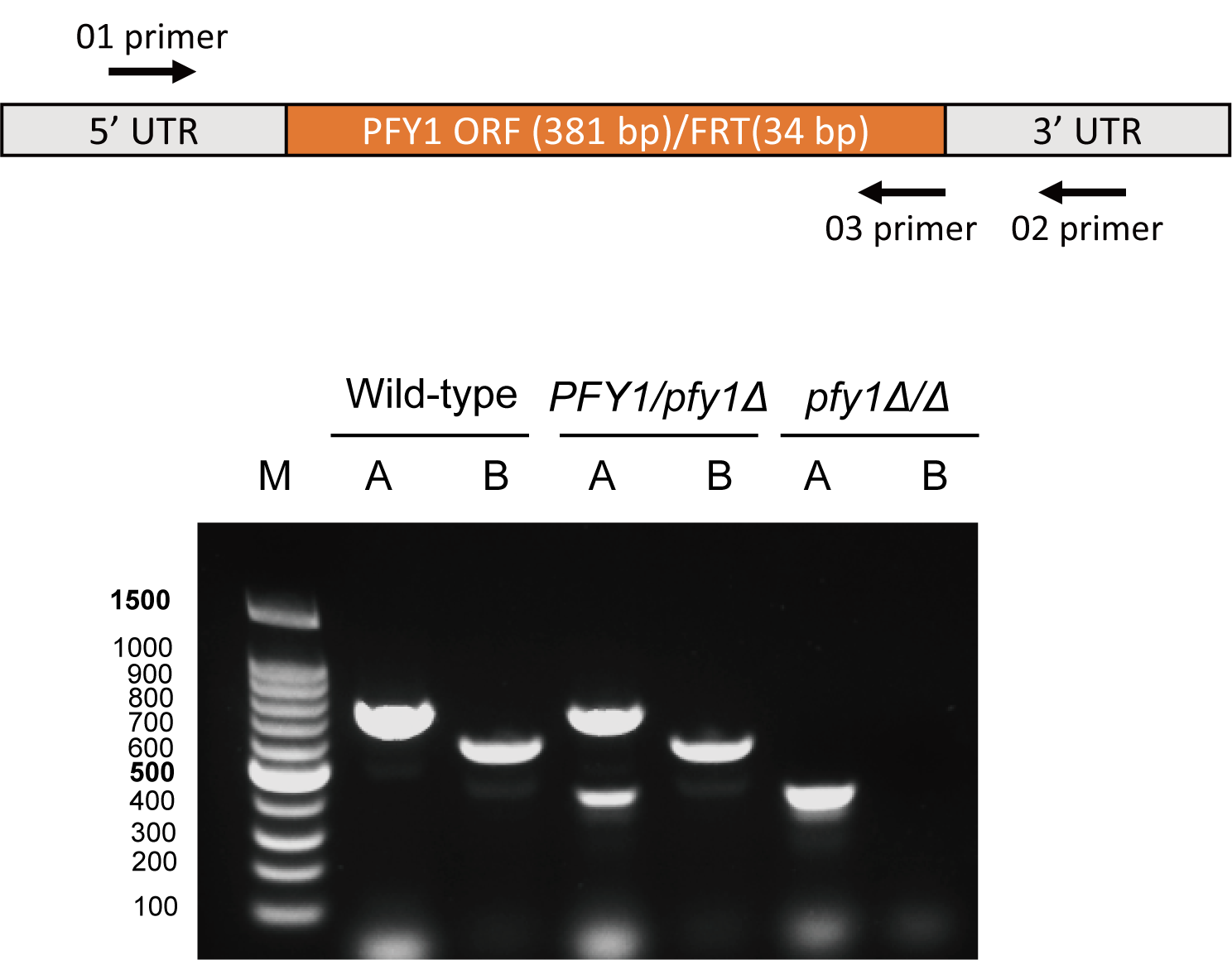
**

**Figure S2. Verification of correct construction of *pfy1Δ/Δ*, *pfy1Δ/Δ+PFY1* strains by PCR**

The PCR primers used are shown on the top. Genomic DNA was extracted from each strain and used as the template for PCR. (A: Primers 01 and 02 , B: Primers 01 and 03)

**
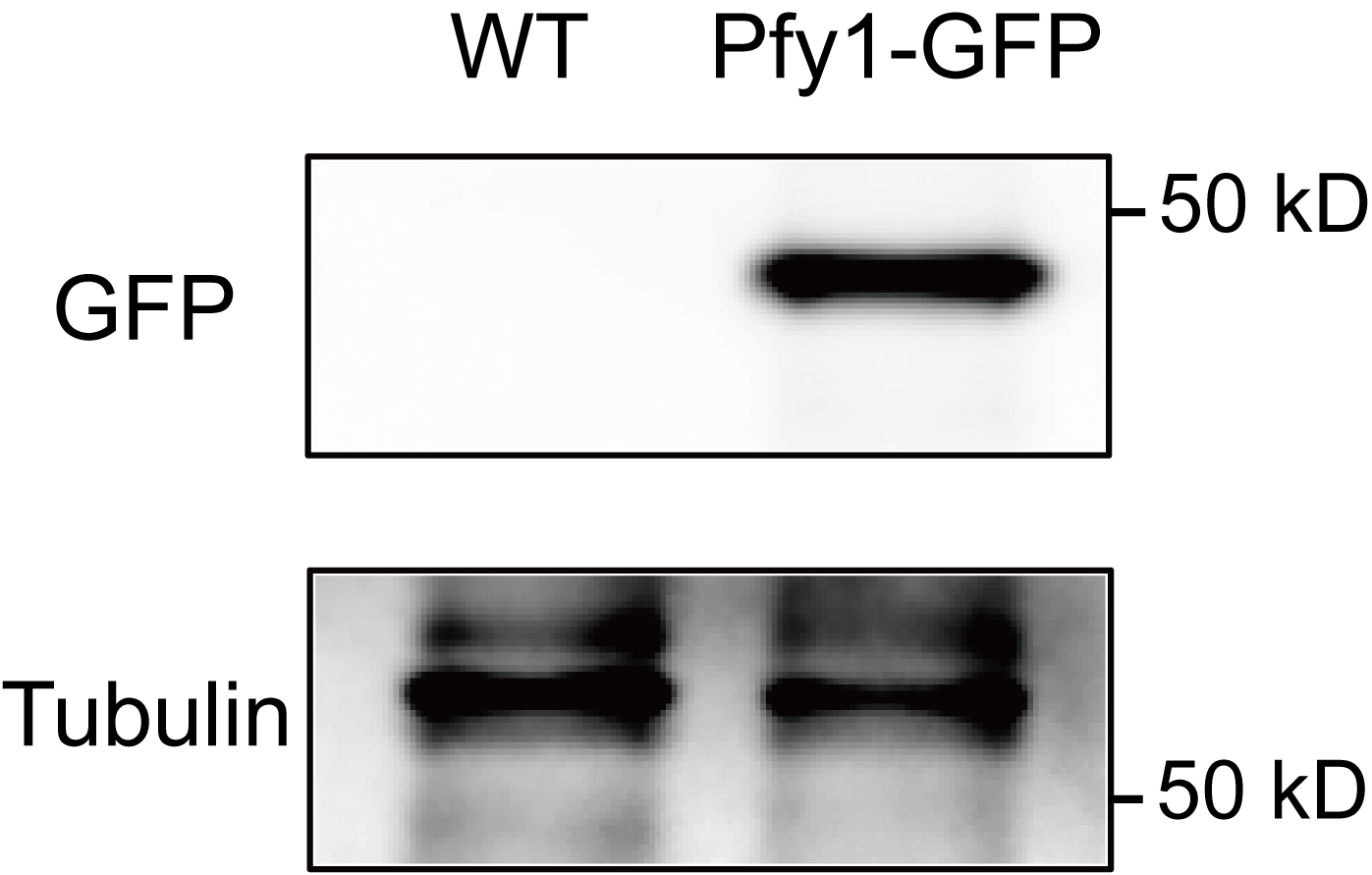
**

**Figure S3. Protein level of Pfy1-GFP was analysis by Western Blot**

Expreesion of the fusion protein was analysis by Western blot. Representative blots of three independent experiments are shown.

**
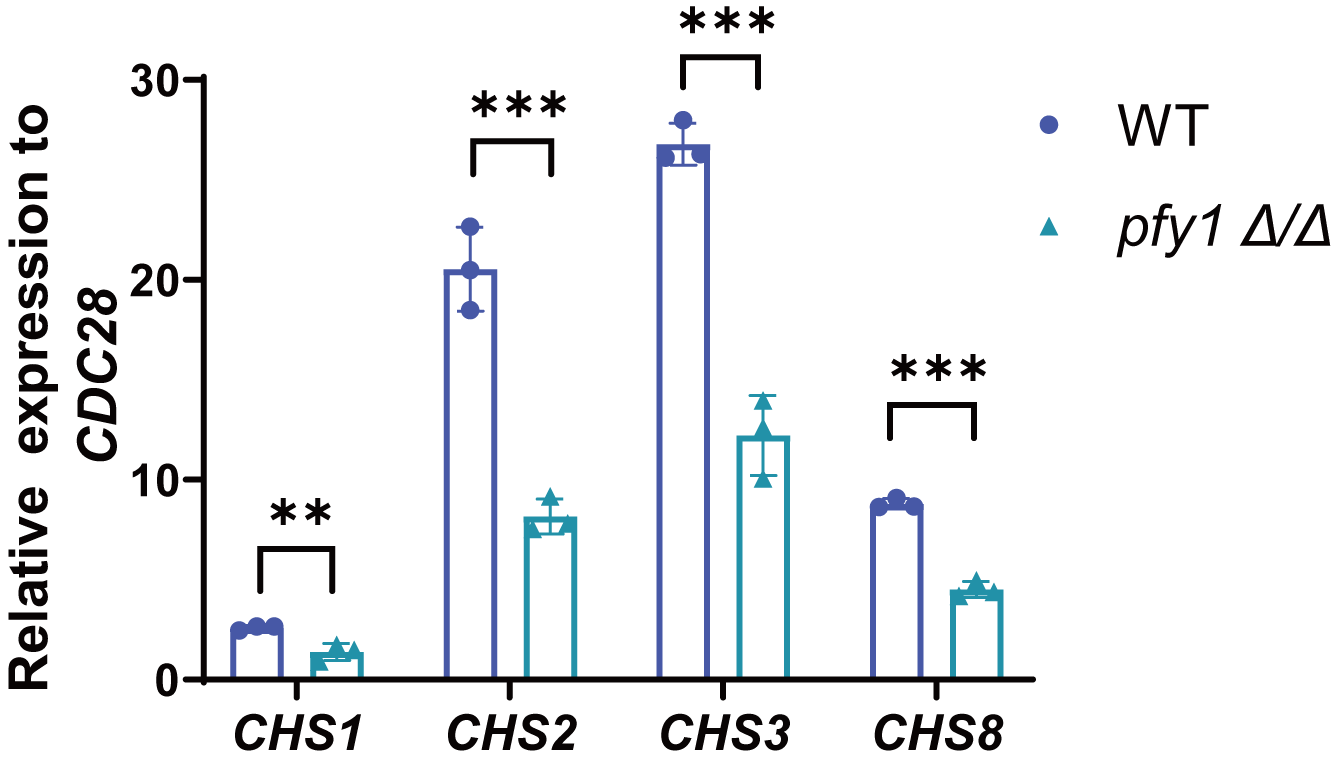
**

**Figure S4. Expression levels of chitin synthase were increased in *pfy1Δ/Δ***

qRT-PCR analysis of expression levels of chitin synthase (*CHS1, CHS2, CHS3,CHS8*) in *pfy1Δ/Δ* and WT cells. Data shown as means ± SD of three independent experiments. Statistical analysis was performed using an unpaired two-tailed Student’s *t*-test. ** represents *p*<0.01; *** represents *p*<0.001.
